# Tenebenal: a meta-diamide with potential for use as a novel mode of action insecticide for public health

**DOI:** 10.1186/s12936-020-03466-4

**Published:** 2020-11-10

**Authors:** Rosemary Susan Lees, Pauline Ambrose, Jessica Williams, John Morgan, Giorgio Praulins, Victoria A. Ingham, Chris T. Williams, Rhiannon Agnes Ellis Logan, Hanafy M. Ismail, David Malone

**Affiliations:** 1 Liverpool School of Tropical Medicine (LSTM), Liverpool, UK; 2 Bill & Melinda Gates Foundation, Seattle, USA; 3 Innovative Vector Control Consortium (IVCC), LSTM, Liverpool, UK

**Keywords:** Insecticide resistance management, *Anopheles gambiae*, *Aedes aegypti*, Meta-diamide, Tenebenal™, Broflanilide

## Abstract

**Background:**

There is an urgent need for insecticides with novel modes of action against mosquito vectors. Broflanilide is a meta-diamide, discovered and named Tenebenal™ by Mitsui Chemicals Agro, Inc., which has been identified as a candidate insecticide for use in public health products.

**Methods:**

To evaluate its potential for use in public health, Tenebenal™ was screened using an array of methodologies against *Anopheles* and *Aedes* strains. Initially it was assessed for intrinsic efficacy by topical application. Tarsal contact bioassays were then conducted to further investigate its efficacy, as well as its potency and speed of action. The potential of the compound for use in indoor residual spray (IRS) applications was investigated by testing the residual efficacy of a prototype IRS formulation on a range of typical house building substrates, and its potential for use in long-lasting insecticidal nets (LLIN) was tested using dipped net samples. Finally, bioassays using well-characterized insecticide-resistant mosquito strains and an in silico screen for mutations in the insecticide’s target site were performed to assess the risk of cross-resistance to Tenebenal™.

**Results:**

Tenebenal™ was effective as a tarsal contact insecticide against both *Aedes* and *Anopheles* mosquitoes, with no apparent cross-resistance caused by mechanisms that have evolved to insecticides currently used in vector control. Topical application showed potent intrinsic activity against a Kisumu reference strain and an insecticide-resistant strain of *Anopheles gambiae.* Applied to filter paper in a WHO tube bioassay, Tenebenal™ was effective in killing 100% of susceptible and resistant strains of *An. gambiae* and *Aedes aegypti* at a concentration of 0.01%. The discriminating concentration of 11.91 µg/bottle shows it to be very potent relative to chemistries previously identified as having potential for vector control. Mortality occurs within 24 h of exposure, 80% of this mortality occurring within the first 10 h, a speed of kill somewhat slower than seen with pyrethroids due to the mode of action. The potential of Tenebenal™ for development in LLIN and IRS products was demonstrated. At least 12 months residual efficacy of a prototype IRS formulation applied at concentrations up to 200 mg of AI/sq m was demonstrated on a range of representative wall substrates, and up to 18 months on more inert substrates. A dipped net with an application rate of around 2 g/sq m Tenebenal™ killed 100% of exposed mosquitoes within a 3-min exposure in a WHO cone test.

**Conclusions:**

Tenebenal™ is a potent insecticide against adult *Aedes* and *Anopheles* mosquitoes, including strains resistant to classes of insecticide currently used in vector control. The compound has shown great potential in laboratory assessment and warrants further investigation into development for the control of pyrethroid-resistant mosquitoes.

## Background

The growing prevalence of insecticide resistance in mosquitoes, and the risk this poses to maintaining the gains made in control of the diseases they transmit, particularly malaria, are well-documented [1, 2]. There is an urgent need to discover and develop compounds with novel modes of action to complement or replace the small number of insecticide classes approved by the World Health Organization (WHO) for use in long-lasting insecticidal nets (LLINs), indoor residual spraying (IRS), larvicides and space sprays [3].

Tenebenal™ (broflanilide), was one of the meta-diamides [*N*-[2-bromo-4-(perfluoropropan-2-yl)-6-(trifluoromethyl)phenyl]-2-fluoro-3-(*N*-methylbenzamido)benzamide] discovered by Mitsui Chemicals Agro Inc. during drug discovery research, a compound with a structure unique among agricultural insecticides and with high activity against several arthropod pest species [4]. Broflanilide was assigned to a new IRAC MoA class 30 (Meta-diamides and Isoxazolines) by the Insecticide Resistance Action Committee (IRAC), due to its novel mode of action [5], as an ionotropic γ-aminobutyric acid (GABA)-gated chloride channel allosteric modulator. Exposure of Lepidoptera to meta-diamides causes symptoms including vomiting and vigorous excitation [4].

The compound is scheduled to be released onto the market in 2020 for agricultural use [4]. Mitsui Chemicals Agro Inc. initiated a collaboration with the Innovative Vector Control Consortium (IVCC) [6] which evaluated the compound for efficacy against mosquito species at the Liverpool Insect Testing Establishment (LITE), Liverpool School of Tropical Medicine (LSTM). IVCC was founded in 2005 with the key objective of facilitating the development of innovative vector control tools, key amongst which are insecticides effective against mosquitoes resistant to pyrethroids [6]. A comprehensive evaluation was performed, and is presented here, involving bioassays for intrinsic activity speed of kill, and potency against *Aedes* and *Anopheles* mosquitoes. The potential of the compound for use in IRS and LLIN vector control products was also evaluated.

Mosquitoes may become resistant to an insecticide as a result of mutations that affect binding of the active ingredient, known as target site resistance. Meta-diamides including Tenebenal™ are antagonists of the GABA receptor, but with binding distinct from non-competitive antagonists (NCAs) such as dieldrin and fipronil [7]. In a previous investigation of broflanilide resistance mechanisms, a screen of mutant Drosophila cell lines found just one mutation which completely abolished the inhibitory effect of meta-diamides, G336M [8]. Mutations in both I277 and L281 were also shown to reduce activity of meta-diamides by around six-fold. Data from the Ag1000G project [9] were screened for point mutations in the target site, which may confer resistance to meta-diamides and isoxazolines, as a measure of whether they exist and are tolerated in target populations of *Anopheles gambiae*. Dose response to Tenebenal™ was determined in well-characterized, insecticide-resistant and -susceptible strains of *An. gambiae* [10] in order to detect any cross-resistance with pyrethroids or other common insecticide classes.

This study is the first to demonstrate the efficacy and potency of Tenebenal™ against mosquito vectors of disease, both in standard laboratory assays and in tests on prototype IRS formulation and dipped nets. Screening genomic data from field populations of *Anopheles* and testing against a range of mosquito strains possessing well-characterized mechanisms of resistance to existing insecticide classes provided no evidence for existing cross-resistance. This study comprises a significant body of evidence for predicted efficacy for Tenebenal™ as well as an evaluation process, which is a model that could be applied when evaluating future novel compounds for their potential use in mosquito vector control.

## Methods

Intrinsic activity of Tenebenal™ against an insecticide susceptible and a characterized resistant strain of *Anopheles* was evaluated through topical application, which bypasses most barriers to uptake by applying a known quantity of active ingredient (AI) directly onto the insect. Efficacy through tarsal contact was evaluated through standard WHO susceptibility bioassays, and speed of kill determined to assess the compound’s speed of action, both against insecticide susceptible and resistant strains.

Tenebenal™ was applied to representative building substrates and tested for residual tarsal activity to evaluate its potential for use in IRS applications. It was also applied to bed net samples to which mosquitoes were then exposed for different periods of time to evaluate its potential for use on LLINs.

Finally, bioassays were performed with well-characterized, insecticide-resistant *An. gambiae* strains to screen for efficacy of the compound against pyrethroid-resistant strains, and the risk of resistance being seen in field populatons assessed in silico by screening *An. gambiae* genome data for mutations in the Tenebenal™ target site.

### Rearing

Mosquito colonies were maintained as described by Williams et al. [10], in the LITE facility at LSTM. Insectary conditions were maintained at 26 ± 2 °C and 80 ± 10% relative humidity (RH), with a L12:D12 hour light:dark cycle and a 1-h dawn and dusk. Larvae were reared in purified water and fed ground TetraMin® tropical flakes (Blacksburg, VA, USA), adults were provided continuous access to a 10% sucrose solution, and adult females given access to blood using a Hemotek membrane feeding system (Hemotek Ltd, Blackburn, UK).

*Anopheles gambiae* Kisumu and *Aedes aegypti* New Orleans are reference insecticide-susceptible strains. Kisumu RDL was selected for dieldrin resistance from the Kisumu strain, and included in this study as it contains a mutation in the γ-aminobutyric acid (GABA) receptor to which Tenebenal™ binds. The Tiassalé 2 strain of *An. gambiae* was colonized from Côte d’Ivoire in 2002, and replaced by a subsequent collection in 2013 to establish the Tiassalé 13 strain; both strains showed resistance to all classes of public health insecticides [10] and were, therefore, included in this study to help assess the cross-resistance risk for Tenebenal™. The *An. gambiae* strain Akron was colonized from Akron in Southern Benin [11], and showed resistance to carbamates, organophosphates and dieldrin [12]. Cayman is an *Ae. aegypti* strain with resistance to DDT and pyrethroids colonized from Grand Cayman in 2008 [13].

Two to five day-old female adult mosquitoes, allowed to mate but not blood-fed, were used in all bioassays.

### Evaluation of intrinsic activity by topical application

A range of concentrations of Tenebenal™ was applied topically to determine its intrinsic activity against adult female *An. gambiae* of the susceptible Kisumu and resistant Tiassalé 2 strains. Three replicates of 10 adult female mosquitoes per treatment were anaesthetized using carbon dioxide and distributed across a Petri dish lined with filter paper (Whatman® Grade 1) on a 4 °C chill table (BioQuip, Rancho Dominguez, CA, USA). A 0.25-µl droplet of technical grade insecticide dissolved in acetone was applied to the dorsal thorax of each mosquito using a 1 cu cm syringe and a hand-operated micro applicator (Burkhard Scientific, Uxbridge, UK). Insecticide treatments consisted of a 0.0001, 0.001, 0.01, 0.1, and 1% of etofenprox and deltamethrin (corresponding to 0.25 to 2,500 pg/mosquito), and 6.25 × 10^–6^, 1.25 × 10^–6^, 2.5 × 10^–5^, 5 × 10^–5^, and 1 × 10^–4^% of Tenebenal™ (15.625 to 250 pg/mosquito; concentrations selected to identify potency relative to deltamethrin). A negative control treatment of acetone-only was also included.

Mosquitoes were assessed for knockdown (KD), defined as being immobile or unable to stand or take-off 30 min after application of the insecticide, then held in cups with access to 10% sucrose at 26 ± 2 °C and 70 ± 10% RH under a L12:D12 hour light: dark cycle inside an environmental stability cabinet for assessment of mortality at 24, 48 and 72 h post-application.

### Evaluation of activity by tarsal contact

#### Tarsal contact assay

The efficacy of Tenebenal™ via tarsal contact was evaluated against 4 mosquito strains (*An. gambiae*: susceptible Kisumu, resistant Kisumu RDL; *Ae. aegypti*: susceptible New Orleans, resistant Cayman) [10]. Solutions of Tenebenal™ were made up in acetone in a tenfold dilution series from 1 to 0.00001%. An aliquot of 0.66 ml of each solution was added to 0.66 ml of a carrier oil (Dow Corning 556 cosmetic grade fluid, Azelis UK Life Sciences Ltd, Hertford, UK) and 0.66 ml of additional acetone. Using a pipette with a disposable tip, the resulting 1.98 ml of mixture was dripped carefully onto each 12 cm × 15 cm filter paper (Whatman® Grade 1), trying to cover the paper as evenly as possible, to give a tenfold dilution series from 366.66 to 0.0036 mg/sq m. Papers were dried overnight in a fume hood. Treated filter papers were wrapped in aluminium foil and stored at 4 °C until used for bioassays.

Three replicates of 10 adult female mosquitoes of each strain were exposed to each filter paper for 60 min using the World Health Organization (WHO) standard susceptibility test method [14], with parallel exposures to an untreated paper and 0.01, 0.1 and 1% etofenprox and deltamethrin papers as reference treatments. After exposure, mosquitoes were held as for the topical application assay and scored for KD (1 h post-exposure) and mortality (24, 48 and 72 h post-exposure).

#### Determining the discriminating dose

The discriminating dose of Tenebenal™ against the susceptible reference strain Kisumu was determined to assess its potency relative to other insecticides, and to provide a dose that can be used to screen field populations for resistance. The discriminating dose (DD) is defined as the LC_95_ multiplied by 3 [15]. To determine the LC_95_ for Tenebenal™, the CDC bottle bioassay [16] was used with some adaptation. Wheaton bottles (250 ml, Fisher Scientific, Loughborough, UK) were coated with 0.01, 0.05, 0.1, 0.25, 0.5, 0.75, 2, 2.5, and 5 µg/bottle Tenebenal™ applied as a solution in acetone and allowed to dry overnight at 26 ± 2 °C and 70 ± 10% RH. Twenty-five female mosquitoes were then exposed per bottle for 1 h under the same conditions. This range of concentrations was shown in range finder assays to result in mortalities between 0 to 100%. An acetone-only bottle and a bottle treated with 20 µg/bottle permethrin dissolved in acetone were included as internal controls to validate each assay; if the mortality in these control bottles was outside the expected range then the assay was rejected and repeated. Mosquitoes were held as described for the topical application assay and mortality scored 24 h after exposure. Data from three replicate bottles, each prepared independently, were used to generate the data used to calculate the LC_95_.

#### Speed of kill assays

Filter papers were treated and mosquitoes exposed as for the tarsal contact assay described above. A single dose of Tenebenal™ was selected for each strain as those needed to provide 100% mortality in each strain based on preliminary dose response experiments: 6.2% for Kisumu, 2.7% for Kisumu RDL, 1.5% for New Orleans, and 5.3% for Tiassalé 2. A negative control blank paper was included as an internal control; if mortality in the control was above 20% then the assay was rejected and repeated. Three replicate papers were tested for each treatment and for the negative control. Mosquitoes were all exposed for 60 min, then maintained under the same conditions as for the previous tarsal contact assay and scored for KD every hour until 12 h post-exposure and for mortality 24, 48 and 72 h post-exposure, to determine how quickly Tenebenal™ kills mosquitoes of different strains post-exposure.

### Testing efficacy and residuality as an IRS formulation

#### Preparation of surfaces

Untreated beech plywood was cut into 12 × 12 cm squares. Readymix cement/concrete (Cemex UK; plasticiser free) was poured into 10-cm diameter Petri dishes and allowed to dry in a climate-controlled stability cabinet (27 ± 2 °C and 80 ± 10% RH) for at least 30 days to produce blocks at least 5 mm thick. Plain glazed ceramic tiles were rinsed in purified water to remove any factory dust or dirt, and allowed to dry prior to treatment. A mud house brick was obtained from partners at Centre Suisse de Recherche Scientifique (CSRS), Côte d’Ivoire and characterized by ACS, Poole, UK (Additional file 1: Table S1). The brick was broken down, sieved, reconstituted with water, and then used to fill 10-cm diameter Petri dishes to produce a smooth mud surface, which was allowed to dry for at least 30 days in a climate-controlled stability cabinet (27 ± 2 °C and 80 ± 10% RH) before treatment and use in bioassays.

#### Treatment of surfaces with IRS formulation

A prototype wettable powder (WP) formulation of Tenebenal™ supplied directly from Mitsui Chemicals Agro Inc. was applied at a rate of 0.2, 0.1, 0.05, and 0.025 g AI/sq m to the prepared surfaces, and residual efficacy against the Kisumu strain of *An. gambiae* tested over 18 months. Surfaces were treated in parallel with Bendiocarb WP (0.4 g AI/sq m), Etofenprox WP (0.1 g AI/sq m) and Deltamethrin WG (0.025 g AI/sq m), and used as reference treatments applied at WHO-recommended rates, and a negative control was treated with water only. Standard IRS formulations were applied at the recommended application rate for vector control, and the range of concentrations of the Tenebenal™ prototype IRS formulation were selected based on the results from the assays described above.

Each treatment was applied to three replicates of each surface type using a ‘Potter Tower’ (Potter Precision Laboratory Spray Tower, Burkard Scientific, Rickmansworth, UK), which applies spray formulations to a 9-cm circle in the centre of the tile, and which corresponds to the area covered by a cone in the WHO cone bioassay [17]. Before test surfaces were treated with the IRS formulation, the centralization of the Potter Tower was validated by demonstrating that the spray rate varied by < 10% across the spray area, the spray weight was validated to vary by < 10% between sprays, and to be within 10% of the target spray weight for the formulation, according to a standard protocol. An additional calibration was performed whereby filter papers were treated with different concentrations of the Tenebenal™ formulation and target dose of the control formulations, and 3 samples were taken from each for high pressure liquid chromatography (HPLC) analysis; with the exception of bendiocarb, distribution was shown to be homogeneous (Additional file 1: Table S2). Surfaces were stored in a climate-controlled stability cabinet at typical conditions under which IRS would be deployed (27 ± 2 °C and 80 ± 10% RH), with air circulation and in the dark, when not in use for bioassays.

#### Cone tests to measure mortality on exposure to IRS-treated surfaces

To measure the residual efficacy of a prototype WP formulation of Tenebenal™, every 4 weeks a WHO cone bioassay [17] was performed with 10 adult female mosquitoes of the susceptible Kisumu strain exposed to all treated surfaces for 30 min. KD was scored 60 min after exposure, and mortality scored 24, 48 and 72 h later, to observe whether the speed of action remained consistent over time, with mosquitoes being held as described for other bioassays between residual efficacy assessments. Bioassays on a given substrate were discontinued if mortality 24 h after exposure was below 80% for two consecutive months, the WHO threshold for IRS efficacy [17]. Occasionally, some bioassays were continued for additional assessments due to month to month variability in mortality.

To confirm that the prototype IRS was also effective against pyrethroid-resistant mosquitoes when applied to different surfaces, resistant Tiassalé 2 strain [10] was exposed to all treated wood surfaces on month 0 and Tiassalé 13 [10] to mud and tile surfaces on months 6 and 8, respectively, to determine the efficacy of the Tenebenal™ WP against characterized resistant strains of *An. gambiae*.

### Testing for potential efficacy on bed nets

#### Treatment of net samples

A suspension concentrate (SC) formulation provided by Mitsui Chemicals Agro Inc., containing 20% Tenebenal™, was applied at 4 concentrations to pieces of polyester netting. Treated net samples were then used in WHO cone bioassays with adult female *An. gambiae* of the Kisumu strain, with a commercially available net containing deltamethrin at 55 mg AI/sq m being used as a positive control treatment and a piece of untreated polyester netting used as a negative control. The volume of solution needed to saturate the net samples without leaving excess liquid (the retention volume) was calculated by immersing a 100-sq cm piece of netting in 100 ml water, wringing out the excess water, and measuring the remaining volume of liquid. This was repeated three times to determine an average volume. Pieces of polyester netting (white, multifilament, 100 deniers) cut into 30 × 30 cm pieces were washed with 1% Decon™ 90 (Decon Laboratories, UK), rinsed thoroughly and dried. The pre-determined retention volume (45.3 ml for 100 sq cm), adjusted for the size (4.08 ml), of each of 4 concentrations (0.28, 1.38, 2.76, 13.80%) was applied in water to the netting pieces in a disposable dish to give final treatment rates of 10, 50, 100 and 500 mg/sq m. Net samples were hung in a fume hood overnight to dry. Samples were cut into 7.5 × 7.5 cm squares, individually wrapped in aluminium foil and kept in a cool place prior to use.

### Chemical analysis to confirm Tenebenal™ application rate on filter paper and net samples

To confirm the accuracy of treatments applied by the Potter tower, filter papers were treated alongside the surfaces with each IRS formulation, and each concentration of Tenebenal™, for subsequent extraction and HPLC analysis of the detected application rates (converted to g/sq m). A 4 × 4 sq cm was cut from the circular area of each sample post-treatment, which was then divided into four 1 sq cm, each of which was placed into a separate tube. Three ml of heptane/1-propoanol solvent mixture (9:1) containing 100 μg/ml of the internal standard dicyclohexyl phthalate (DCP, Sigma Aldrich UK) was added to each tube, and sonicated for 30 min. One ml of the extract was evaporated to dryness under nitrogen at 40˚C, resuspended in 1 ml of methanol and 500 μl transferred to an Eppendorf tube and centrifuged at 12,000×*g* for 15 min. The insecticide content of each sample was determined using HPLC by injecting 10 μl aliquots of the extract on a reverse-phase Hypersil GOLD C18 column (175 Å, 250 × 4.6 mm, 5 μl, Thermo Scientific, UK) at 23–25˚C, setting the mobile phase of acetonitrile/water 93:7 (optimize for each analyte) at a flow rate of 1 ml/min for a period of time optimized for each analyte to separate insecticide and DCP. Analyte peaks were detected at a wavelength of 232 nm, except for Tenebenal™ which was detected at 210 nm, and the quantities of insecticide and DCP calculated from standard curves established by known concentrations of analytical grade standards, in μg/ml.

To confirm the application rate on net samples, insecticide was extracted in triplicate from net pieces with an area of 48 sq cm according to Toé et al. [18]. The insecticide content of each sample was determined using HPLC by injecting 10 μl aliquots of the extract on a reverse-phase Hypersil GOLD C18 column with detailed HPLC conditioned mentioned in filter paper analysis section.

### Cone tests to measure mortality on exposure to dipped net samples

WHO cone bioassays were performed as described for the IRS assay using three replicate net samples for each treatment and the negative control, with 10 mosquitoes exposed in each of replicates per treatment. Mosquitoes were exposed initially for 3 min (as stated in the WHO guidelines [17]) but this was then increased to an exposure period of 20 min. KD was scored at 60 min after the start of exposure, and mortality scored 24, 48 and 72 h post-exposure.

### Testing for cross-resistance

#### Calculating resistance ratios in vivo

To look for evidence of any cross-resistance to Tenebenal™ in mosquitoes resistant to existing classes of insecticide, LC_50_ values were calculated using data from WHO susceptibility bioassay on resistant strains and resistance ratios calculated relative to the LC_50_ values for susceptible strains. Kisumu, Kisumu RDL and Tiassalé 2 were exposed to 8 concentrations of Tenebenal™ applied to filter papers in a WHO susceptibility bioassay treated as for the tarsal contact assay described above: 0.0625, 0.125, 0.25, 0.5, 0.75, 1, 1.5 and 5%. Mortality was scored at 24 h, and data from at least 3 replicates of 25 female adults were used to calculate the LC_50_. The LC_50_ values were then used to calculate resistance ratios relative to the susceptible strain, Kisumu.

#### Screening for target site mutations in silico

The *Anopheles gambiae* 1000 genome database (Ag1000G) [9], containing at the time of this study the full genome sequences from 1,142 individuals, was interrogated to identify variation in the GABA-gated chloride channel gene, *Rdl*. Additional file 2: Table S3 details all non-synonymous mutations in the gene.

### Data analysis

Percentage KD and mortality were calculated from the total number of mosquitoes in each replicate of the topical and tarsal assays counted at the end of the experiments. If mortality in the topical application or tarsal contact test negative control was between 5 and 20%, mortality in test replicates was corrected using Abbott’s formula [19], with negative values reported as 0% mortality. If control mortality was above 20% at 24 h after exposure the test was repeated. Breakpoints were determined as being the lowest concentration at which 80% or more mosquitoes were killed at each observation post-exposure.

The lethal concentration (LC) values of Tenebenal™ were calculated using PoloPlus software (Version 2.1, LeOra Software), via log-probit analysis [20], for 24-h mortality data from at least two replicates of approximately 25 mosquitoes exposed to each concentration. The number of subjects (n) and the number of responders (r) were aggregated across three replicates of each concentration tested, comprising the highest concentration tested that gave ~ 0% mortality and the lowest that gave ~ 100% mortality, and an even range of doses between these. The calculated LC_95_ value for the Kisumu strain was multiplied by 3 to give a discriminating dose (LC_95_ × 3 = DD), as previously defined by Lees et al*.* [15]. LC_50_ values are reported with 95% confidence intervals and Chi square analysis, calculated using PoloPlus software (Version 2.1, LeOra Software). Resistance ratios were calculated by dividing the LC_50_ of each resistant strain by the LC_50_ of the susceptible comparator strain.

## Results

### Topical application assay for intrinsic activity

When topically applied to mosquitoes from the pyrethroid-susceptible Kisumu strain of *An. gambiae*, Tenebenal™ gave a high level of rapid KD and kill at all concentrations tested, reaching 80% mortality 24 h after application above 0.000025% and at lower concentrations when scored at 48 or 72 h (Table 1). The insecticidal effect on mosquitoes was rapid, with very high levels of KD 30 min after application. This level of efficacy and potency was in the same range as for deltamethrin, which gave 100% KD or kill at all time points.

**Table 1:** Effect of topical application of Tenebenal™ and two standard comparators on adult female *Anopheles gambiae*

Strain	Insecticide	Concentration	30 min		24 h		48 h		72 h	
			KD (%)	Breakpoint (%)	Mortality (%)	Breakpoint (%)	Mortality (%)	Breakpoint (%)	Mortality (%)	Breakpoint (%)
Kisumu	Tenebenal™	0.00000625%	100.0	< 0.00000625	66.7	0.000025	60.0	0.00000125	63.3	0.00000125
		0.00000125%	88.0		73.3		80.0		80.0	
		0.000025%	88.4		83.9		96.8		100.0	
		0.00005%	100.0		96.7		100.0		100.0	
		0.0001%	91.7		96.6		96.6		96.6	
	Etofenprox	0.0001%	87.1	< 0.0001	50.0	0.001	53.6	0.001	53.6	0.001
		0.001%	95.9		82.8		89.7		86.2	
		0.01%	96.0		93.3		96.7		96.7	
		0.10%	100.0		100.0		100.0		100.0	
		1%	100.0		100.0		96.7		96.7	
	Deltamethrin	0.0001%	100.0	< 0.0001	100.0	< 0.0001	100.0	< 0.0001	100.0	< 0.0001
		0.001%	100.0		100.0		100.0		100.0	
		0.01%	100.0		100.0		100.0		100.0	
		0.10%	100.0		100.0		96.6		100.0	
		1%	100.0		100.0		100.0		100.0	
Tiassalé 2	Tenebenal™	0.00000625%	6.7	> 0.0001	96.7	< 0.00000625	96.7	< 0.00000625	96.7	< 0.00000625
		0.00000125%	36.7		96.7		100.0		100.0	
		0.000025%	17.4		90.0		96.7		96.7	
		0.00005%	18.5		97.0		100.0		100.0	
		0.0001%	23.3		86.7		100.0		100.0	
	Etofenprox	0.0001%	11.1	1	14.4	1	14.4	0.1	14.4	0.1
		0.001%	56.7		13.3		26.7		23.3	
		0.01%	71.9		51.9		62.2		58.5	
		0.1%	66.7		70.0		80.0		80.0	
		1%	89.3		92.6		92.6		92.6	
	Deltamethrin	0.0001%	65.9	0.001	13.7	0.1	16.7	0.01	20.0	0.01
		0.001%	85.6		57.4		46.7		46.7	
		0.01%	74.1		74.1		85.2		96.3	
		0.1%	90.0		100.0		100.0		100.0	
		1%	96.7		100.0		100.0		100.0	

Efficacy was tested against Kisumu (insecticide susceptible) and Tiassalé 2 (resistant) strains. KD and mortality was observed 30 min and 24, 48 and 72 h after topical application, respectively, and were corrected for negative control mortality using Abbott’s formula where mortality was greater than 5%. The breakpoint at each observation point denotes the lowest concentration at which the 80% mortality threshold, the measure of efficacy given by WHO for IRS formulations, was reached

In the insecticide-resistant Tiassalé 2 strain, there was a lower rate of KD immediately after exposure to Tenebenal™ than in the susceptible Kisumu females, but mortality reached the 80% threshold at the lowest concentration tested, 0.00000625%. The lower rate of mortality with deltamethrin and etofenprox reference controls confirmed the known pyrethroid resistance of this strain.

### Evaluation of activity by tarsal contact

#### Tarsal contact assay

Mortality when taken up tarsally was measured by exposing mosquitoes to dried residues of Tenebenal™ on a glass petri dish, and in this assay the compound was slower, giving low rates of KD 60 min after exposure (Table 2). However, mortality was above 80% 24 h after exposure (the ‘breakpoint’) to concentrations above 0.001% (0.3667 mg/sq m) in all strains except for the resistant Cayman *Ae. aegypti* strain. In Cayman, the 80% mortality threshold was reached at a concentration of 0.01% (3.667 mg/sq m) by 24 h post-exposure, but when observed 48 h post-exposure the threshold was reached at the lower concentration (0.001%). There is also some evidence for delayed mortality in Kisumu, where the breakpoint was lower when observed after 48 h post-exposure than after 24 h, and lower still after 72 h.

**Table 2:** Effect of exposure to Tenebenal™ applied to a filter paper in a WHO tube bioassay on adult female mosquitoes

Strain	Concentration (%)	% Knock down or Mortality				Breakpoint 24 h	Breakpoint 48 h	Breakpoint 72 h
		1 h	24 h	48 h	72 h			
Kisumu (*An. gambiae*)	1	0.0	100.0	100.0	100.0	0.001	0.0001	< 0.00001
	0.1	4.0	100.0	100.0	100.0			
	0.01	0.0	100.0	100.0	100.0			
	0.001	10.0	100.0	100.0	100.0			
	0.0001	6.7	76.0	88.0	92.0			
	0.00001	0.0	39.3	67.9	82.1			
Kisumu RDL (*An. gambiae*)	1	0.0	100.0	100.0	100.0	0.001	0.001	0.001
	0.1	3.2	100.0	100.0	100.0			
	0.01	0.0	100.0	100.0	100.0			
	0.001	0.0	100.0	100.0	100.0			
	0.0001	0.0	65.4	68.9	49.8			
	0.00001	0.0	0.0	25.0	11.0			
Akron (*An. gambiae*)	1	5.6	100.0	100.0	100.0	0.001	0.001	0.001
	0.1	0.0	100.0	100.0	100.0			
	0.01	5.6	100.0	100.0	100.0			
	0.001	0.0	100.0	100.0	100.0			
	0.0001	0.0	0.0	5.6	16.7			
	0.00001	10.5	16.7	55.6	61.1			
Tiassalé 2 (*An. gambiae*)	1	0.0	100.0	100.0	100.0	0.001	0.001	0.001
	0.1	0.0	100.0	100.0	100.0			
	0.01	0.0	88.9	91.8	100.0			
	0.001	0.0	100.0	100.0	100.0			
	0.0001	0.0	30.0	14.8	20.8			
	0.00001	2.2	31.3	27.1	29.5			
New Orleans (*Ae. aegypti*)	1	0.0	100.0	100.0	100.0	0.001	0.001	0.001
	0.1	10.0	100.0	100.0	100.0			
	0.01	0.0	96.7	100.0	100.0			
	0.001	30.0	100.0	100.0	100.0			
	0.0001	20.0	26.7	33.3	33.3			
	0.00001	20.0	33.3	33.3	33.3			
Cayman (*Ae. aegypti*)	1	0.0	100.0	100.0	100.0	0.01	0.001	0.001
	0.1	0.0	100.0	100.0	100.0			
	0.01	0.0	100.0	100.0	100.0			
	0.001	0.0	79.3	89.7	96.6			
	0.0001	0.0	3.6	7.1	14.3			
	0.00001	0.0	0.0	0.0	3.3			

KD and mortality was observed 1, 24, 48 and 72 h after the start of exposure, and is reported as the average of 3 replicates of 10 mosquitoes. Values are corrected for negative control mortality using the Abbott’s formula where mortality in the negative control was greater than 5%. In one replicate of the Tiassalé 2 assay one negative control tube produced 100% mortality; this replicate was excluded from analysis. The breakpoint at each observation point denotes the lowest concentration at which the 80% mortality threshold, the measure of efficacy given by WHO for IRS formulations [17], was reached

Positive controls gave 100% KD and kill for all *Anopheles* strains. Etofenprox killed all New Orleans *Ae. aegypti* strain mosquitoes by the end of exposure, and 44, 50 and 70% of resistant Cayman *Ae. aegypti* strain mosquitoes by 72 h post-exposure. Deltamethrin killed 70, 100 and 90% of New Orleans exposed to 0.01, 0.1 and 1% papers, respectively, by 72 h post-exposure, and all exposed Cayman by the end of exposure.

#### Determining the discriminating dose

**Fig. 1:**
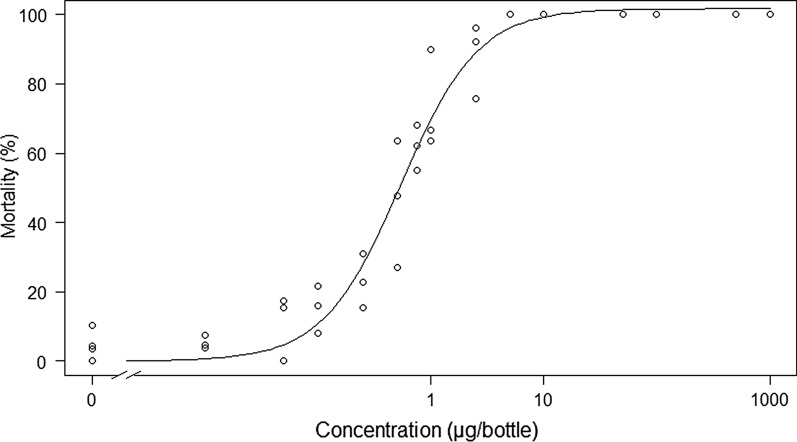
The 24-h mortality dose response curve of female *Anopheles gambiae* adults of the susceptible Kisumu strain exposed to Tenebenal™ in a CDC bottle bioassay

The dose response curve for Tenebenal™ in susceptible Kisumu *An. gambiae* exposed in a CDC bottle bioassay is shown in Fig. 1. The LC_50_ was calculated as 0.58 µg/bottle (95% confidence intervals 0.444–0.723), the LC_90_ as 2.6 µg/bottle (95% confidence intervals 1.938–4.113), and the LC_95_ as 3.97 µg/bottle (95% confidence intervals 2.751–7.206). The discriminating dose (defined as three times the LC_95_) was calculated as 11.91 µg/bottle.

#### Speed of kill

Since the tarsal contact experiment indicated that most mosquitoes affected by TENENEBAL™ were knocked down between 1 and 24 h post-exposure, an experiment was conducted to determine the speed of action for the compound, that is the time taken for mosquitoes to be killed post-exposure.

**Fig. 2:**
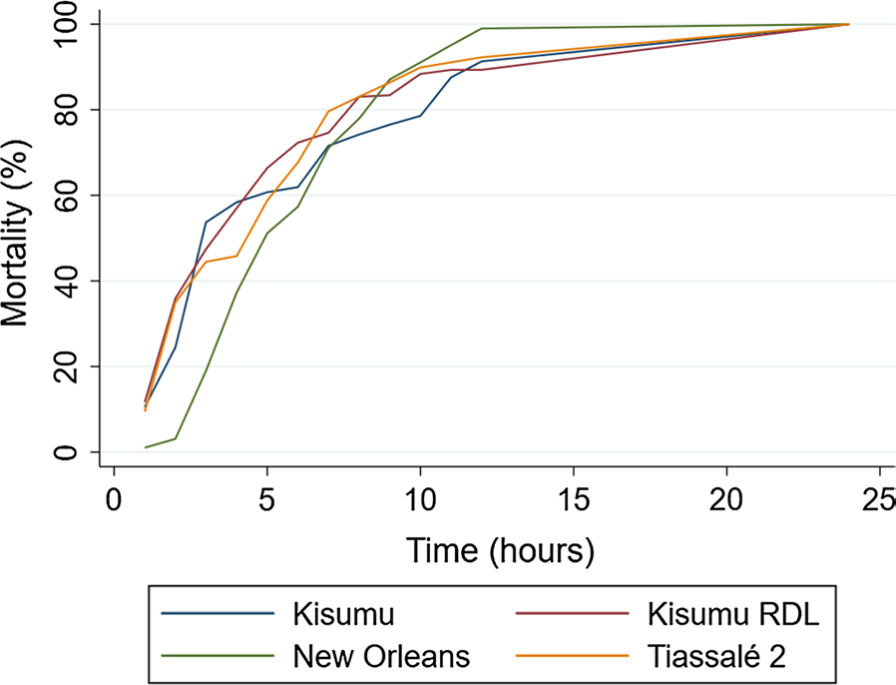
Mortality over time in four mosquito strains after exposure to Tenebenal™ applied to a filter paper in a WHO tube bioassay. Concentrations used for each strain were those needed to provide 100% mortality in each strain based on preliminary dose response experiments (not presented), and were as follows: Kisumu 6.2%, Kisumu RDL 2.7%, Tiassalé 2 5.3%, and New Orleans 1.5%. Mosquitoes were scored for KD or mortality every hour until 12 h and then at 24 h post-exposure. Results are presented as an average of three replicate tubes of ~ 25 mosquitoes.

Because of the mode of action of Tenebenal™, mosquitoes were not expected to be killed immediately after tarsal exposure, so the speed of activity was tested by exposing mosquitoes in a WHO tube susceptibility bioassay with KD being scored every hour up to 12 h, and then mortality scored at 24, 48 and 72 h post-exposure. High mortality within 1 h of exposure to deltamethrin confirmed the pyrethroid-susceptible status of the susceptible New Orleans, RDL and Kisumu strains, and similarly substantial recovery from initial KD confirmed the resistance status of Tiassalé 2. Tenebenal™ knocked exposed mosquitoes down more slowly than deltamethrin, and in the effect on the New Orleans *Ae. aegypti* strain was slower than in the other strains for the first 3 to 4 h post-exposure, although mortality was 100% in all strains within 24 h (Fig. 2), the WHO definition of IRS efficacy.

### Testing efficacy and residuality as an IRS formulation

#### HPLC analysis of filter papers and net samples to confirm application rates

The amount of insecticide delivered to filter papers treated using the Potter Tower alongside the bioassay surfaces, and to polyester net samples by dipping, was quantified using HPLC analysis and compared to the target application rate (Table 3). In all cases there was an over-application of insecticide, most notably in the case of bendiocarb filter papers and the dipped net samples where the application rate was nearly 6 times the intended rate in some cases. Since the distribution of the bendiocarb IRS was also very heterogeneous in the Potter Tower calibration, the observed over-treatment might be a result of the formulation having a fast precipitation rate and so being unevenly distributed by the Potter Tower.

**Table 3:** Application rate of insectide formulations applied to filter papers and polyester net samples

Treatment	Target application rate	Average detected rate		Difference (%)
	(mg of AI/sq m)	(mg of AI/sq m)	Relative Standard Deviation	
Filter papers				
Tenebenal™ WP	200	281	N/A	+ 40.5
	100	125	N/A	+ 25
	50	63	N/A	+ 26
	25	29	N/A	+ 16
Etofenprox WP	100	156	N/A	+ 56
Bendiocarb WP	40	272	N/A	+ 580
Deltamethrin WG	25	37	N/A	+ 48
ITNs treated with Tenebenal™				
Tenebenal™ WP	10	70	40	+ 595
	50	265	31	+ 430
	100	401	15	+ 301
	500	2188	25	+ 338
Control net	Control	ND	ND	ND

N/A: one filter paper was treated per concentration so relative standard deviation could not be calculated. ND: no analysis done on untreated control net sample

#### Cone tests to measure mortality on exposure to IRS-treated surfaces

**Fig. 3:**
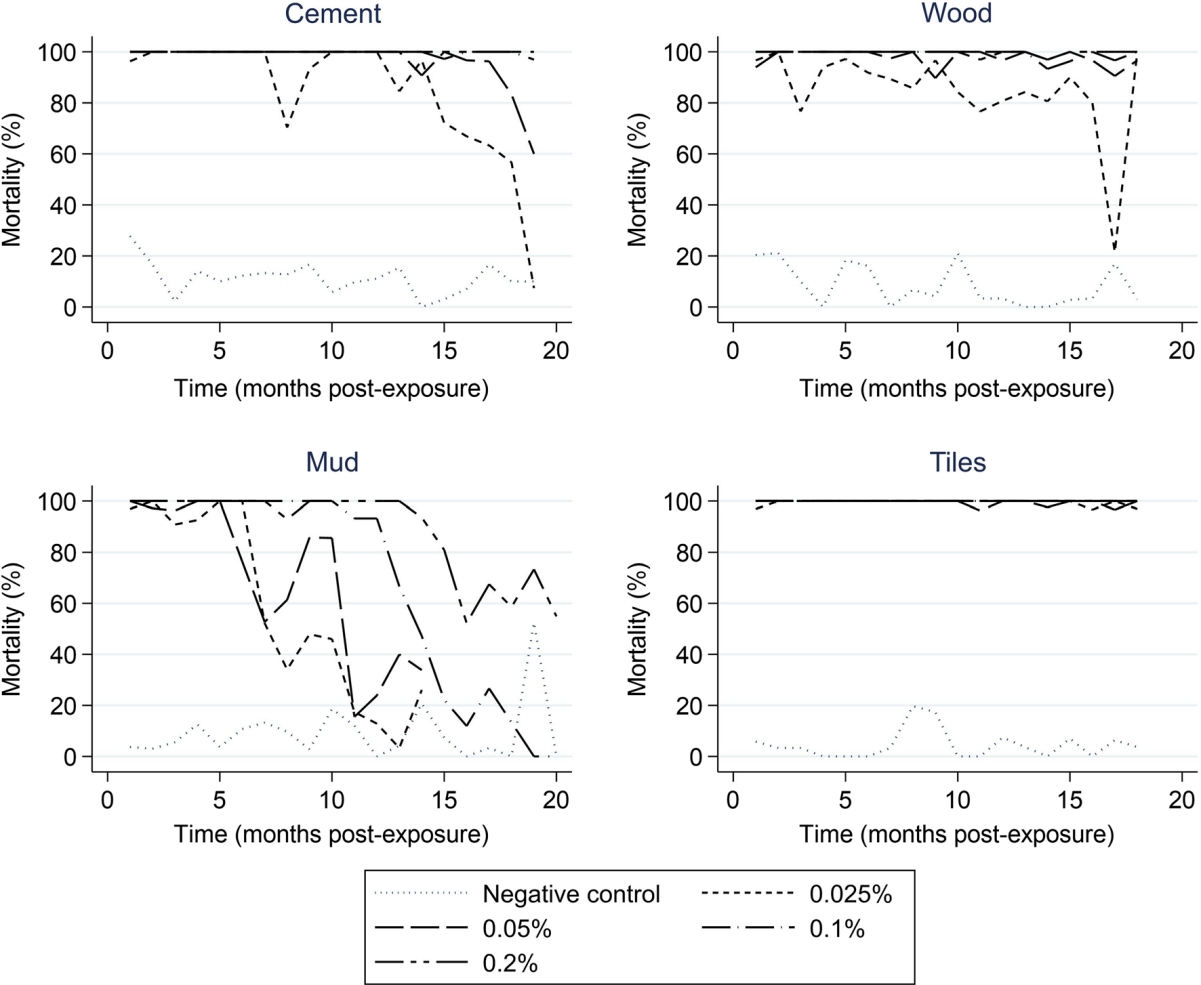
Residual activity of Tenebenal™ WP applied at four concentrations as a prototype WP IRS to four surface materials, in comparison to an untreated control, measured as mortality in adult female *Anopheles gambiae* of the Kisumu strain 24 h after exposure. Mosquitoes were exposed to mud, cement, ceramic tiles or wood treated with 0.025, 0.05, 0.1 or 0.2% solutions of Tenebenal™ WP or an untreated negative control, and mortality observed 24 h after exposure. Values shown are average mortality in 30 mosquitoes, 10 exposed to each of three replicate surfaces. Mud blocks with 0.025 and 0.05% were withdrawn after month 14 due to low activity, but assays were continued with higher concentrations until month 20. Mortality in the negative control ceramic tile for month 10 was anomalously high but returned to expected value at subsequent time points; this datapoint is omitted from the Figure

The WHO requirement for efficacy of an IRS treatment is ≥ 80% mortality of mosquitoes following a 30-min exposure to a treated substrates in a WHO cone bioassay and a 24-h holding period, and/or 95% KD [17]. When applied to ceramic tiles, the Tenebenal™ WP formulation killed more than 95% of exposed mosquitoes at all concentrations for the whole 18 months duration of the experiment (Fig. 3). On wood and cement, Tenebenal™ WP remained active, as defined by the WHO as ≥ 80% (15), for 18 months at all concentrations with the exception of the lowest concentration 0.025% where mortality fell below 80% in months 3, 11 and 17 on wood, and in month 8 and from month 15 to the end of the experiment on cement. On mud blocks, residual efficacy was maintained for the first 5 months, after which mortality decreased in a dose-dependent manner, with the highest concentration, 0.2%, killing only ~ 60% of exposed mosquitoes by the end of the experiment.

Bioassays were discontinued on mud blocks treated with 0.025 and 0.05% Tenebenal™ WP from month 15 onwards, as efficacy had remained below the 80% threshold for several months. At month 18, bioassays were discontinued on ceramic tiles and on cement and wood blocks, because the efficacy had been demonstrated for the designated period, but assays on remaining mud surfaces were continued until month 20 as results were variable and further information could be collected.

When mortality was scored 48 h after exposure, activity was maintained for 18 months at all concentrations when applied to ceramic, wood and cement tiles, and for 10 months on mud blocks at the lowest two concentrations, 12 months at 0.1% and 20 months at 0.2% (Additional file 1: Fig. S1). Observed at 72 h, mortality dropped below 80% on cement in months 18 and 19 but activity was retained for the whole experiment on wood and ceramic tiles; on mud, efficacy was maintained for 12 months at the lowest two concentrations, 17 months at 0.1% and the full 20 months at 0.2% (Additional file 1: Fig. S2). KD of susceptible Kisumu mosquitoes immediately after exposure did not consistently reach the 95% threshold on any surface (Additional file 1: Fig. S3).

In contrast, the bendiocarb WP only reached the 80% mortality threshold for 2 months on cement and did not reach this threshold on any other surface even in the first month post-treatment, even though the actual application rate was far higher than the recommended field rate of 0.4 g AI/sq m. Deltamethrin WG and etofenprox WP reached the 80% mortality threshold for residual efficacy for 1 and 4 months, respectively, on mud, for 13 months on cement, and for the full 18 months on tiles and wood. Control data are shown in Additional file 1: Fig. S4.

**Fig. 4:**
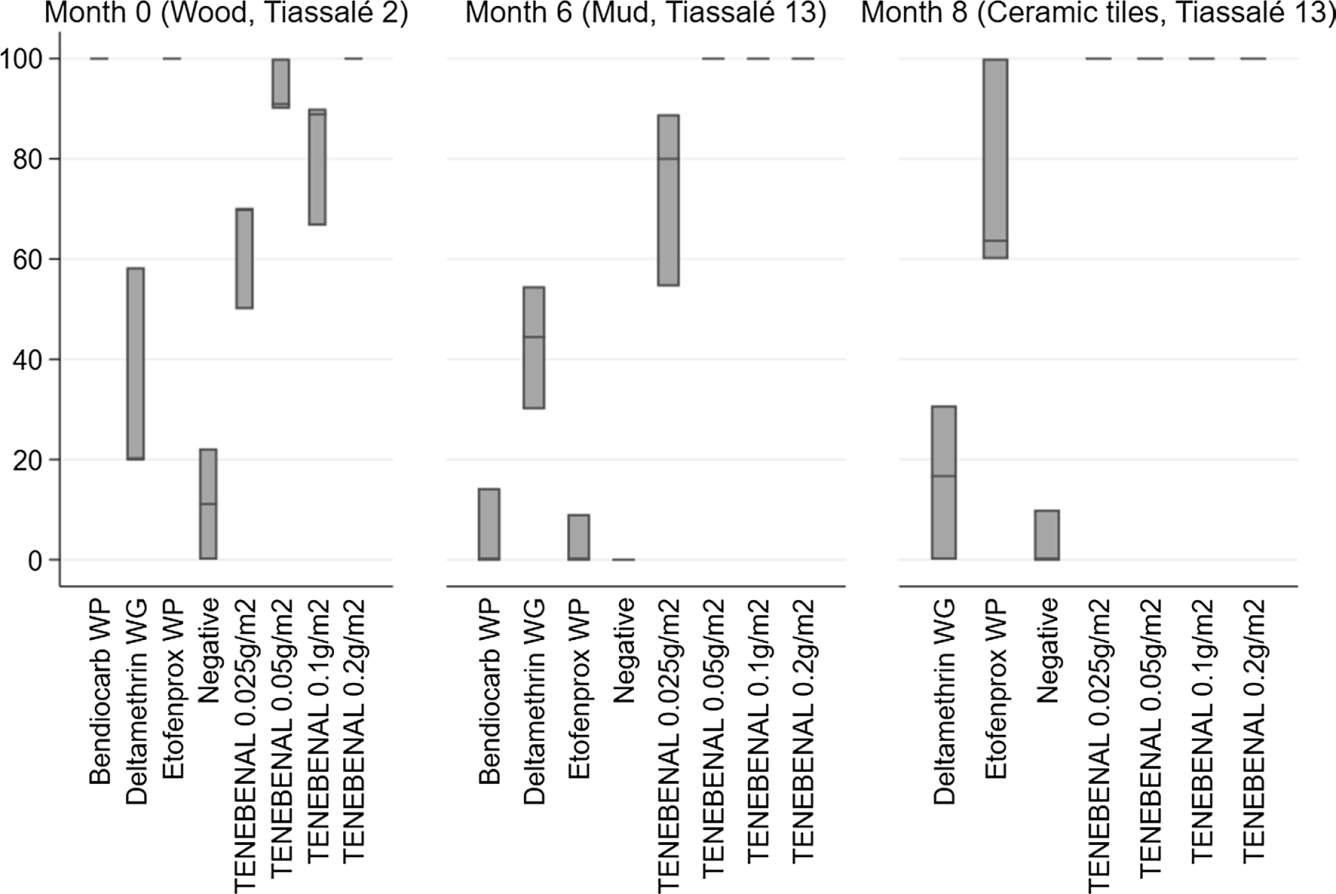
Residual activity of Tenebenal™ applied in four concentrations as a prototype WP, as measured by cone bioassays, in comparison to three standard IRS products and an untreated negative control, against insecticide resistant strains of *Anopheles gambiae.* Mosquitoes were exposed to surfaces for 30 min, and mortality observed 24 h after exposure: Tiassalé 2 females were exposed to treated wood surfaces on the day after treatment, and Tiassalé 13 females were exposed to treated mud surfaces and ceramic tiles in months 6 and 8 post-treatment application, respectively. Values shown are average mortality in 30 mosquitoes, 10 exposed in cones to each of three replicate tiles; replicates are represented by the top, dissecting line, and bottom of each bar

To assess the efficacy of the Tenebenal™ WP formulation against pyrethroid-resistant mosquitoes, the Tiassalé 2 strain of *An. gambiae* were exposed to the treated wood surfaces alongside the first round of WHO cone bioassays. The resistance profile was confirmed by the low mortality caused by deltamethrin-treated surfaces (Fig. 4). Tenebenal™ WP killed 100% of the resistant mosquitoes at the highest concentration (0.2 g/sq m), and above 80% when applied at 0.05 and 0.1 g/sq m.

To assess the residual efficacy of Tenebenal™ WP against pyrethroid-resistant mosquitoes, cone bioassays were performed on treated mud surfaces at month 6 and ceramic tiles at month 8 post-treatment application using the Tiassalé 13 strain, a more recently colonized strain established from mosquitoes collected from the same site as the Tiassalé 2 strain (Fig. 4). The low residual efficacy of standard IRS formulations on ceramic tiles, which were still potent against the susceptible Kisumu strain, demonstrates the high level of resistance to existing insecticide classes. By contrast, 100% of adults were killed by all Tenebenal™ WP-treated surfaces, except for the lowest concentration applied to mud surfaces where mortality was 74%.

### Testing for potential efficacy on bed nets

#### Cone tests to measure mortality on exposure to dipped net samples

**Fig. 5:**
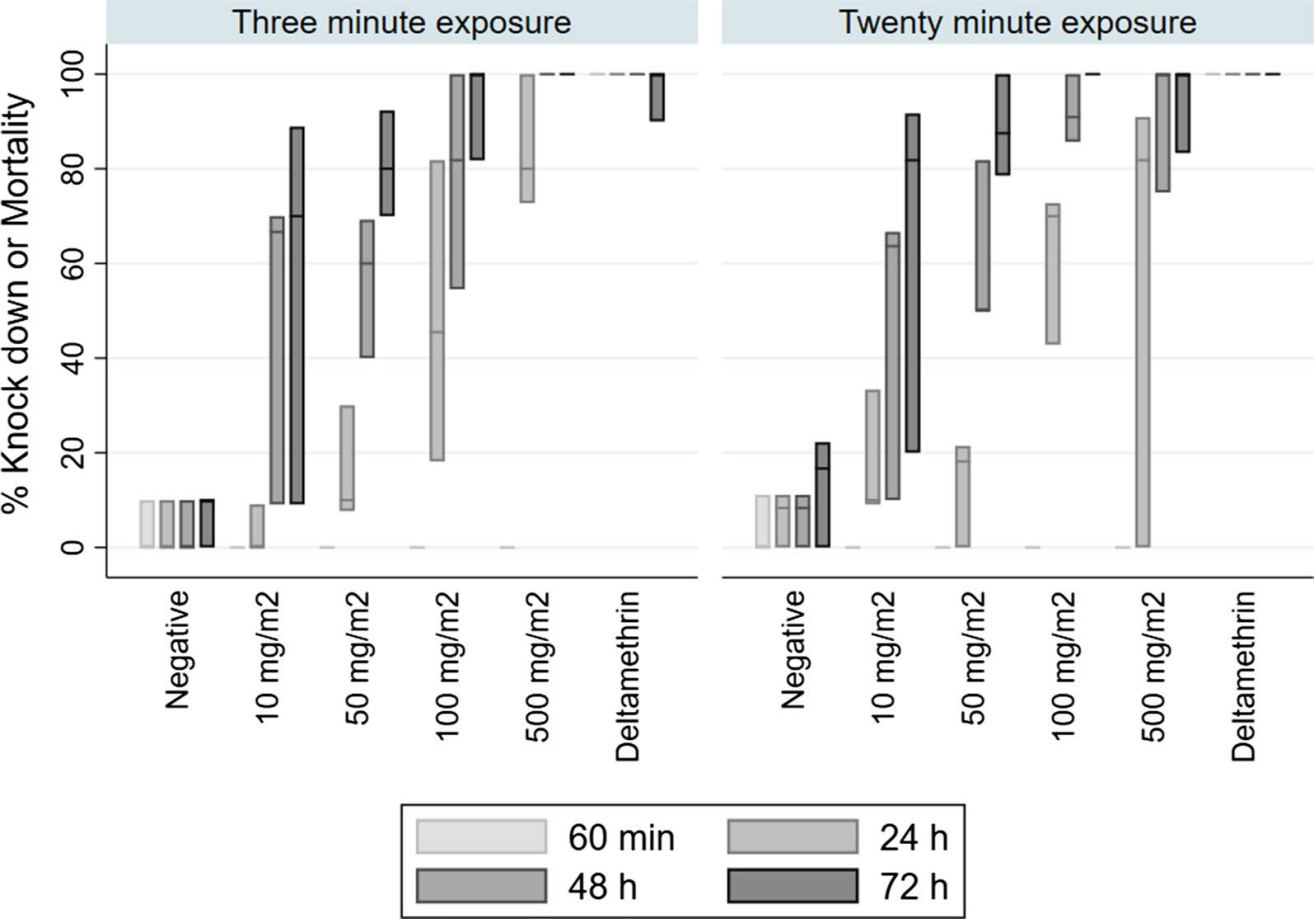
Assessment of the efficacy of net samples dipped in Tenebenal™ SC using cone bioassays, in comparison to a standard deltamethrin net and an untreated negative control, against adult female Kisumu *Anopheles gambiae.* Mosquitoes were exposed to surfaces for 3 or 20 min, KD observed 60 min later, and mortality observed 24, 48 and 72 h after exposure. Values shown are average mortality in 30 mosquitoes, 10 exposed per cone to each of the three replicate net samples; replicates are represented by the top, dissecting line, and bottom of each bar

To test whether Tenebenal™ might be effective on an ITN, Kisumu mosquitoes were exposed to dipped net samples in a WHO cone assay. The WHO defines efficacy of an LLIN as ≥ 80% mortality after 24 h or ≥ 95% KD 60 min after exposure for 3 min in a cone bioassay [21]. No KD was measured in mosquitoes exposed to the Tenebenal™ SC-treated netting (Fig. 5). Mortality reached the ≥ 80% threshold 24 h post-exposure at the highest concentration tested following a 3-min exposure in cones, a concentration approximately 10 times greater than the concentration of deltamethrin in the control net sample, but only reached this threshold 48 or 72 h post-exposure at the lower concentrations. Exposing female adults to the net samples for 20 min only slightly increased the observed mortality.

### Cross resistance liability

#### Calculating resistance ratios in vivo

The LC_50_ values for each strain (and 95% CI) calculated using mosquitoes from each strain exposed to filter papers with a range of concentrations in a WHO tube assay were as follows: Kisumu 0.11% (0.067–0.16%, Chi^2^ = 24.46, df = 6, p < 0.001), Kisumu RDL 0.19% (0.156–0.232%, df = 6, Chi^2^ = 17.20, df = 6, p < 0.01), New Orleans 0.14% (0.131–0.157%, Chi^2^ = 3.24, p_[0.05, df=6]_ = 3.24) and Tiassalé 2 0.37% (0.258–0.518%, Chi^2^ = 43.06, df = 6, p < 0.001). Kisumu RDL and Tiassalé 2 were, therefore, significantly (p < 0.05) more tolerant to the Tenebenal™ insecticide than the standard susceptible strain Kisumu, but New Orleans was not. The resistance ratios were 1.73 for Kisumu RDL, 1.27 for New Orleans and 3.36 for Tiassalé 2. These small resistance ratios likely arise from differences in the fitness of the cohorts of mosquitoes used for this experiment, leading to a slightly higher tolerance. Further repetition would help to confirm this, but there is at least no evidence for a dramatically reduced mortality in resistant strains compared to Kisumu.

#### Screening for target site mutations in silico

Three amino acids have previously been indentified that disrupt the efficacy of Tenebenal™ binding: G336, I277 and L281 corresponding to G331, I272 and L276 in *An. gambiae*. These amino acids surround the binding pocket of Tenebenal™ and any mutations in natural *Anopheles* vectors at these positions could result in resistance to this compound. To determine the risk of target site resistance in field populations, the Phase 2 Ag1000 data was screened for all variation, including non-synonymous mutations (Additional file 2: Table S3). No mutations were found in G331, I272 or L276, indicating that resistance to this compound is unlikely to occur in field populations.

## Discussion

This study is the first to demonstrate the efficacy of Tenebenal™, indeed any meta-diamide pesticide, against mosquito vectors of disease. The efficacy of the compound via tarsal contact against *An. gambiae* and *Ae. aegypti* mosquitoes, including strains with resistance to multiple classes of insecticide, and an absence of point mutations in the target site in *Anopheles* genomic data suggests a low risk of cross-resistance when used against field populations. The potential for the use of Tenebenal™ for use in IRS or insecticide-treated bed net formulations is demonstrated. A prototype wettable powder formulation showed comparable residual efficacy when compared with existing WHO PQ-VCT listed IRS formulations [22]. The evidence presented here from laboratory bioassays supports the further evaluation of both LLIN and IRS formulations containing Tenebenal™ in the laboratory, and if successful in the field, against locally dominant mosquito vector populations in relevant settings.

The results from all laboratory bioassays performed suggest that Tenebenal™ is an effective and highly potent insecticide against adult mosquitoes. The concentrations used in the topical application assay were orders of magnitude lower than were used in the same protocol to screen compounds for repurposing as public health insecticides (0.01–1%) [15]. This previous screen used a glass plate tarsal contact assay method which differs from the standard WHO susceptibility bioassay where the mosquitoes are exposed to a treated filter paper instead, making direct comparison of potency by tarsal contact difficult. However, the concentrations applied per unit area in each assay were approximately equivalent, and the breakpoint in 24-h mortality in Kisumu was approximately tenfold lower for Tenebenal™ than for clothianidin, the most potent compound identified by the screen (12). As assessed by discriminating dose, Tenebenal™ is more potent than all insecticides previously identified by IVCC as having potential for repurposing for use against malaria vectors, apart from clothianidin [15], and the LC_50_ of Tenebenal™ against the susceptible Kisumu strain of *An. gambiae* was very similar.

As a proof of concept, a prototype WP formulation of Tenebenal™ was produced by Mitsui Chemicals Agro Inc. and applied to a range of substrates representative of building materials to which an IRS product might be applied for mosquito vector control. Residual efficacy was demonstrated for the full 18 months of testing on some surfaces, and for 12 months on the more challenging surfaces. Thus, the potential for long-term stability of a preliminary Tenebenal™ WP formulation on an inert surface was demonstrated. Given the measured over-application of formulation to the surfaces measured by HPLC, the concentration required in any final IRS formulation would require further investigation. The known challenges of achieving long-lasting residual efficacy on a relatively uneven and porous mud surface (for example [23, 24]) was again observed and will need to be a consideration in developing a final IRS formulation. Even though a preliminary prototype IRS formulation of Tenebenal™ was used to measure residual efficacy, it performed well compared to existing IRS products applied at their recommended application rate. A final formulated product is therefore expected to be a powerful new IRS product, and given its new mode of action a valuable addition to the range of IRS products available for use in rotation to minimize the development of resistance.

Tenebenal™ is a pro-insecticide which must be metabolized into the active form within the insect [8], and the minimal KD observed immediately after exposure and increased residual efficacy of IRS formulation when mortality was scored beyond the standard 24-h observation period suggests that it is slower acting than pyrethroids used in vector control. The WHO define efficacy in IRS as ≥ 80% mortality within 24 h of exposure to a treated surface. Using this definition Tenebenal™ can be reported as active in this study for at least 18 months on ceramic, wood and cement tile substrates at all but the lowest concentration tested (0.025%). For some active ingredients being developed for use as IRS against mosquitoes, most notably those which are pro-insecticides, it is proposed that mortality should be scored later, due to their novel mode of action being slower acting than the pyrethroids for which the WHO guidelines were developed [25, 26]. When applied to mud surfaces, the speed of action seemed to decrease with time post-treatment, suggestive of a dose-dependent speed of action. Extending the definition of efficacy to include those mosquitoes which died up to 72 h after exposure would extend the reported residual efficacy from 5 to 12 months, and for longer at the higher concentrations tested.

Cone bioassays with net samples dipped in an SC formulation demonstrates that Tenebenal™ also has potential for development into an LLIN product. Dipped net samples with an application rate of around 2 g/sq m Tenebenal™ (in an SC formulation) gave > 80% mortality with a 3-min exposure; for comparison, the Olyset® net (Sumitomo Chemical Co. Ltd.) contains 1 g/sq m permethrin, the Interceptor® net (BASF SE) contains 0.2 g/sq m alpha-cypermethrin, and the Interceptor G2® net (BASF SE) 0.1 mg/sq m α-cypermethrin and 0.2 g/sq m chlorfenapyr. Again, further investigation is required to determine the effective dose on an ITN, and this small experiment serves as only a proof of principle. In contrast to the deltamethrin-dipped nets used in this study, no KD was observed with the Tenebenal™ SC dipped net samples. Chloride channel-blocking insecticides typically cause hyperexcitability and convulsions in exposed insects, but as a pro-insecticide Tenebenal™ may be slower acting that pyrethroids when presented on a net, in common with other compounds being applied to the newer LLINs. A bed net reduces malaria transmission in two ways. Community protection is offered when mosquitoes are killed on contact. Personal protection is offered by a rapid KD, which prevents the person sleeping under the net from being bitten, and may be helped by some measure of repellency and blood-feeding inhibition. These potential additional effects have yet to be investigated for Tenebenal™. To maximize the efficacy of an ITN in preventing malaria transmission a combination of insecticides may be applied, for example a slow-acting insecticide may be combined with a faster-acting compound. The first such net to reach field deployment is the Interceptor G2® which contains both chlorfenapyr and the pyrethroid α-cypermethrin. The other benefit of this approach is that combined use of insecticides with different modes of action is consistent with good insecticide resistance management.

Tenebenal™ binds to a target site in the insect γ-aminobutyric acid (GABA) receptor that is distinct from that affected by RDL mutations, and which leads to resistance to non-competitive antagonists (NCAs) such as dieldrin [8]. Mutations in the target site which confer resistance have been identified in Drosophila [7], but it is perhaps not surprising that they were not detected in individual *An. gambiae* sampled from wild populations since they have not yet been exposed to Tenebenal™. Given the fitness costs associated with target site mutations [27, 28], the risk of target site resistance to Tenebenal™ occurring in natural *Anopheles* populations in the absence of selective pressure would be expected to be low [7]. It is possible that other sites may be important in conferring resistance, or that multiple mutations co-existing may be significant, as in the case of kdr/s-kdr mutants, where kdr must first be present for s-kdr to be functionally permissive [29]. There is no evidence so far for existing cross-resistance in the pyrethroid-resistant laboratory strains resulting from more general resistance mechanisms, such as over-expression of cytochrome P450s metabolic enzymes [30], cuticular thickening [31] or over-expression of chemosensory proteins [32]. Using data from the genome databases is an effective and efficient way to look for resistance liability in a population, allowing measurement of baseline prevalence of resistance markers as well as heterogeneity at the sites important for binding of an insecticide prior to deployment. The use of multiple, well-characterized, resistant strains of mosquitoes allows this to be further investigated in vitro. Further investigation would be required to rule out the possibility that these mechanisms, or behavioural resistance [33], may result in cross-resistance.

In addition to target site mutations, resistance to an insecticide may be conferred by upregulation of detoxification genes [30], reduced penetration of the cuticle [34], or upregulation of pyrethroid binding proteins [32, 35]. These mechanisms may not be specific to pyrethroid resistance, and may confer cross-resistance to different insecticide classes. It is therefore important for potential new vector health insecticides to be screened against well-characterized resistant strains carrying a range of resistance mechanisms [10]. The efficacy of Tenebenal™ against mosquito strains resistant to multiple classes of insecticide currently in use for public health is demonstrated by several bioassay methods. The potency of the compound was greater against the resistant strain Tiassalé 2 than the susceptible Kisumu by topical application, but the KD scored immediately after exposure was higher in Kisumu strain than Tiassalé 2 strain. Further investigation would be needed to determine the mechanism for this difference, which may be a result of metabolic differences between the two strains affecting the speed of activation of the pro-insecticide or of differential speeds of uptake. The speed of kill after tarsal exposure was not significantly different between strains, and potency in the tarsal contact assay was similar between strains of *An. gambiae*, as measured from 24-h mortality, which may point to a slower tarsal uptake of the compound but this would need further confirmation. The 48- and 72-h mortality was slightly higher in Kisumu at the lowest concentrations tested, and there is a small difference between resistant Cayman and susceptible New Orleans *Ae. aegypti* strains, although this may be an artefact of variability in the assay results. The slightly slower activity against *Ae. aegypti* may be a result of differences in tarsal uptake rate between mosquito genera: this is supported by the higher breakpoints observed in the tarsal contact assay. However, all mosquitoes were killed by 12–24 h after exposure, as required by the WHO guidelines for assessing the efficacy of new IRS products [17]. Although the calculated LC_50_ values were significantly different between Kisumu RDL and Tiassalé 2 resistant strains and the susceptible comparator Kisumu, the resistance ratios were only 1.73 and 3.36, respectively. Resistance ratios for pyrethroids against resistant strains of *Anopheles* maintained in the LITE facility ranged from 11–384 when measured using the same methodology [10], suggesting that any difference in susceptibility to Tenebenal™ is not biologically significant. Furthermore, surfaces treated with the prototype IRS formulation were effective against both susceptible and resistant strains of *An. gambiae* up to 8 months post-treatment.

## Conclusions

Tenebenal™ is the first novel insecticide to be developed with the support of the IVCC programme, and represents a new mode of action and IRAC class [5] for public health use. The compound’s potency has been demonstrated both through direct topical application and through tarsal contact. The compound demonstrates the potential for development into both a LLIN and long-lasting IRS formulation, although its slow action may require that Tenebenal™ be combined with an insecticide with a faster mode of action on a dual AI-treated net both for personal protection and for insecticide resistance management purposes. No target site mutations were detected in screened genome data, and during testing with well-characterized pyrethroid resistant strains of *Aedes* and *Anopheles* mosquitoes no evidence of cross-resistance was found. Indeed, data suggest that binding of Tenebenal™ to the GABA receptor is unaffected by the Rdl mutation present in the Kisumu RDL strain. These findings indicate that Tenebenal™ has the potential for use in the control of resistant populations of *Anopheles*. Offering a completely novel mode of action, the compound may play a key role in insecticide resistance management.

## Supplementary information

**Additional file 1: Table S1.** Physico-chemical properties of the mud house brick used to make the testing tiles (ACS, Poole, UK). **Table S2.** Results of Potter Tower calibration exercise to investigate distribution of application of active ingredient across filter papers treated with IRS formulations using HPLC analysis of active ingredient content across the paper. **Fig. S1.** Residual activity of TenebenalTM WP applied in four concentrations as a prototype WP IRS to four surface materials, in comparison to an untreated control, measured as mortality in adult female Anopheles gambiae of the Kisumu strain 48 hours after exposure. **Fig. S2.** Residual activity of TenebenalTM WP applied in four concentrations as a prototype WP IRS to four surface materials, in comparison to an untreated control, measured as mortality in adult female Anopheles gambiae of the Kisumu strain 72 hours after exposure. **Fig. S3.** Residual activity of TenebenalTM WP applied in four concentrations as a prototype WP IRS to four surface materials, in comparison to an untreated control, measured as knock down in adult female Anopheles gambiae of the Kisumu strain observed immediately after exposure. **Fig. S4.** Residual efficacy of three standard IRS formulations applied to four substrates, in comparison to an untreated control, measured as mortality in adult female Anopheles gambiae of the Kisumu strain 24 hours after exposure.

**Additional file 1: Table S3.** Ag1000 frequencies for all non-synonymous mutations in the Rdl GABA receptor.

## Data Availability

The datasets generated and/or analysed during the current study are not publicly available due to commercial sensitivities but are available from the corresponding author on reasonable request.

## Abbreviations

LLIN: Long Lasting Insecticidal Nets
IRS: Indoor Residual Sprays
GABA: γ-Aminobutyric acid
AI: Active Ingredient
WHO: World Health Organization
IVCC: Innovative Vector Control Consortium
LITE: Liverpool Insect Testing Establishment
IRAC: Insecticide Resistance Action Committee
LSTM: Liverpool School of Tropical Medicine
NCAs: Non-Competitive Antagonists
KDR: Knock Down Resistance
CDC: Centre for Disease Control
DD: Discriminating Dose
LC: Lethal Concentration
HPLC: High-performance Liquid Chromatography
WP: Wettable Powder
WG: Wettable Granule
W/V: Weight by volume
RDL: Resistant to Dieldrin
