# Supplementary material for: Tenebenal: a meta-diamide with potential for use as a novel mode of action insecticide for public health
Source: Malar J. 2020 Nov 10;19:398. doi: 10.1186/s12936-020-03466-4 (PMC7654575; doi:10.1186/s12936-020-03466-4)
Supplement: Supplementary file 1 — Additional file 1: Table S1. Physico-chemical properties of the mud house brick used to make the testing tiles (ACS, Poole, UK). Table S2. Results of Potter Tower calibration exercise to investigate distribution of application of active ingredient across filter papers treated with IRS formulations using HPLC analysis of active ingredient content across the paper. Fig. S1. Residual activity of TenebenalTM WP applied in four concentrations as a prototype WP IRS to four surface materials, in comparison to an untreated control, measured as mortality in adult female Anopheles gambiae of the Kisumu strain 48 hours after exposure. Fig. S2. Residual activity of TenebenalTM WP applied in four concentrations as a prototype WP IRS to four surface materials, in comparison to an untreated control, measured as mortality in adult female Anopheles gambiae of the Kisumu strain 72 hours after exposure. Fig. S3. Residual activity of TenebenalTM WP applied in four concentrations as a prototype WP IRS to four surface materials, in comparison to an untreated control, measured as knock down in adult female Anopheles gambiae of the Kisumu strain observed immediately after exposure. Fig. S4. Residual efficacy of three standard IRS formulations applied to four substrates, in comparison to an untreated control, measured as mortality in adult female Anopheles gambiae of the Kisumu strain 24 hours after exposure. [file 12936_2020_3466_MOESM1_ESM.docx]

**Additional file 1: Table S1. Physico-chemical properties of the mud house brick used to make the testing tiles (ACS, Poole, UK)**

| **Property** | **Sample A** | **Sample B** |
| --- | --- | --- |
| **Elemental analysis** | | |
| Aluminium | 1.86% | 1.95% |
| Calcium | 0.034% | 0.028% |
| Iron | 0.67% | 0.70% |
| Potassium | 0.12% | 0.13% |
| Magnesium | 0.035% | 0.037% |
| Sodium | 0.015% | 0.015% |
| Titanium | 0.031% | 0.032% |
| Arsenic | 0.0022% | 0.0023% |
| Cadmium | < 0.0001% | < 0.0001% |
| Chromium | 0.0025% | 0.0026% |
| Copper | 0.0011% | 0.0012% |
| Mercury | < 0.0001% | < 0.0001% |
| Nickel | 0.0006% | 0.0007% |
| Lead | 0.0002% | 0.0002% |
| Selenium | Nil | Nil |
| Zinc | 0.0025% | 0.0020% |
| Manganese | 0.0078 | 0.0083 |
| Phosphorus | 0.0027 | 0.0026 |
| Sulphur | 0.0065 | 0.0061 |
| Vanadium | 0.0021 | 0.0019 |
| Barium | 0.0075 | 0.0078 |
| Silica | ND | ND |
| **Properties** | | |
| Organic matter | 0.52% | 0.57% |
| Acid soluble sulfate | 0.070% | 0.050% |
| CEC (cation exchange capacity) | 8.31 | 8.38 |
| Acid soluble chloride | 0.030% | 0.040% |
| pH | 8.1 | 7.10 |
| Water soluble chloride | 16.5mg/L | 15.1mg/L |
| Water soluble fluoride | 0.07mg/L | 0.06mg/L |
| Water soluble phosphate | <0.02 | <0.02 |
| Water soluble sulfate | 11.6 | 9.96 |
| Water soluble nitrate | 6.11 | 5.52 |
| Water soluble nitrite | 0.2 | 0.1 |
| **Physical properties** | | |
| Optimum moisture content (%)* | 10 | 9 |
| Dry density (mg/cu m) | 1.8 | ND |
| Plastic limit (%) ^ | 12 | 12 |
| Liquid limit (%) $ | 21 | 20 |
| % passing 425 µ | 95 | 94 |
| Porosity calculated TW (% voids) | 31 | ND |
| **Composition** | | |
| Cobbles (60-200 mm) (%) | 0 | 0 |
| Gravel (2-60 mm) (%) | 0 | 3 |
| Sand (63 µ-2mm) (%) | 49 | 44 |
| Silt (2-63 µ) (%) | 34 | 35 |
| Clay (<2 µ) (%) | 17 | 18 |
|  |  |  |
| * The water content at which a maximum dry unit weight can be achieved after a given compaction effort. | | |
| ^ Determined by rolling out a thread of the fine portion of a soil on a flat, non-porous surface. | | |
| $ The water content at which the behaviour of a clayey soil changes from the plastic state to the liquid state. | | |
| **Additional file 1: Table S2 Results of Potter Tower calibration exercise to investigate distribution of application of active ingredient across filter papers treated with IRS formulations using HPLC analysis of active ingredient content across the paper.**   \| **Active ingredient (AI)** \| **Sample Name** \| **Target (mg AI/sq m)** \| **Average (mg AI/sq m)** \| **N** \| **STDEV** \| **RSD** \| \| --- \| --- \| --- \| --- \| --- \| --- \| --- \| \| \| \| Tenebenal**^TM^** \| MA \| 125 \| 129.8 \| 3 \| 17.6 \| 13.6 \| \| Tenebenal**^TM^** \| MB \| 250 \| 249.0 \| 3 \| 21.3 \| 8.6 \| \| Tenebenal**^TM^** \| MC \| 500 \| 530.9 \| 3 \| 23.1 \| 4.4 \| \| Tenebenal**^TM^** \| MD \| 1000 \| 1175.2 \| 3 \| 59.7 \| 5.1 \| \| Bendiocarb \| B \| 500 \| 471.6 \| 3 \| 159.6 \| 33.8 \| \| Deltamethrin \| D \| 50 \| 69.4 \| 3 \| 12.2 \| 17.6 \| \| Etofenprox \| E \| 200 \| 196.5 \| 3 \| 42.3 \| 21.5 \|   **Additional file 1: Table 3 Ag1000 freqeuncies for all non-synonymous mutations in the Rdl GABA receptor**  Chromsome, chromosomal position, reference allele, SNP, nucleotide change and position and the amino acid change and position is shown for each non-synonymous SNP, frequencies are then shown by column for each country. | | |


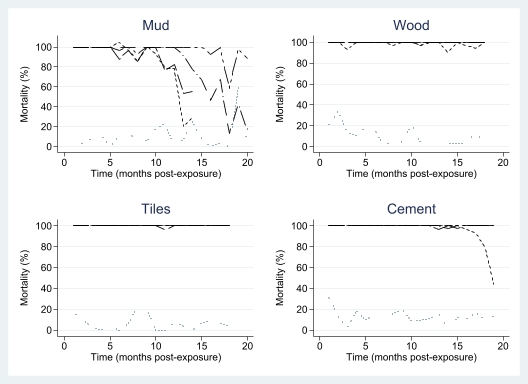


**Additional file 1: Fig. S1 Residual activity of Tenebenal^TM^ WP applied in four concentrations as a prototype WP IRS to four surface materials, in comparison to an untreated control, measured as mortality in adult female *Anopheles gambiae* of the Kisumu strain 48 hours after exposure**

Mosquitoes were exposed to mud, cement, ceramic tiles or wood treated with 0.025, 0.05, 0.1 or 0.2% solutions of Tenebenal**^TM^** WP or an untreated negative control, and mortality observed 24 hours after exposure. Values shown are average mortality in 30 mosquitoes, 10 exposed to each of three replicate tiles. Mud tiles with 0.025 and 0.05% were withdrawn after month 14 due to low activity, but assays were continued with higher concentrations until month 20. Mortality in the negative control ceramic tile for month 10 was anomalously high but returned to expected value at subsequent time points; this datapoint is omitted from the Figure.

**Additional file 1: Fig. S2 Residual activity of Tenebenal^TM^ WP applied in four concentrations as a prototype WP IRS to four surface materials, in comparison to an untreated control, measured as mortality in adult female *Anopheles gambiae* of the Kisumu strain 72 hours after exposure**

Mosquitoes were exposed to mud, cement, ceramic tiles or wood treated with 0.025, 0.05, 0.1 or 0.2% solutions of Tenebenal**^TM^** WP or an untreated negative control, and mortality observed 24 hours after exposure. Values shown are average mortality in 30 mosquitoes, 10 exposed to each of three replicate tiles. Mud tiles with 0.025 and 0.05% were withdrawn after month 14 due to low activity, but assays were continued with higher concentrations until month 20. Mortality in the negative control ceramic tile for month 10 was anomalously high but returned to expected value at subsequent time points; this datapoint is omitted from the Figure.

**Additional file 1: Fig. S3 Residual activity of Tenebenal^TM^ WP applied in four concentrations as a prototype WP IRS to four surface materials, in comparison to an untreated control, measured as knock down in adult female *Anopheles gambiae* of the Kisumu strain observed immediately after exposure**

Mosquitoes were exposed to mud, cement, ceramic tiles or wood treated with 0.025, 0.05, 0.1 or 0.2% solutions of Tenebenal**^TM^** WP or an untreated negative control, and mortality observed 24 hours after exposure. Values shown are average mortality in 30 mosquitoes, 10 exposed to each of three replicate tiles. Mud tiles with 0.025 and 0.05% were withdrawn after month 14 due to low activity, but assays were continued with higher concentrations until month 20.


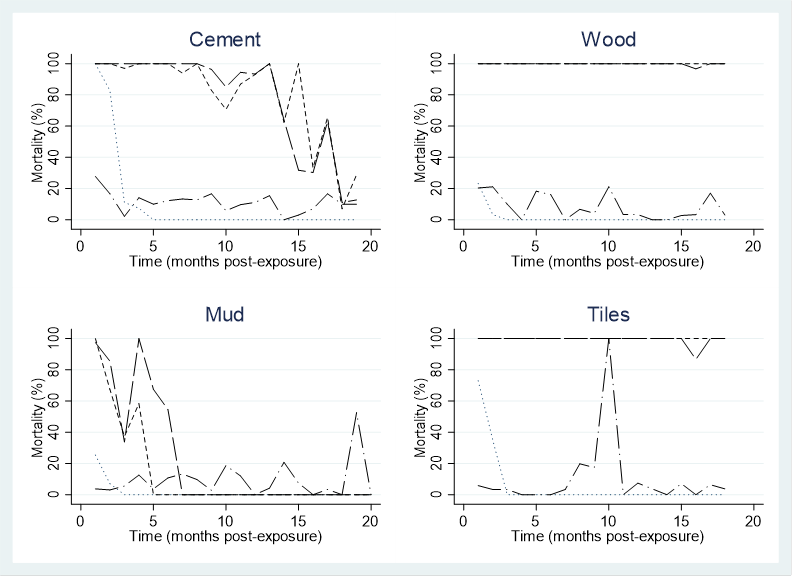


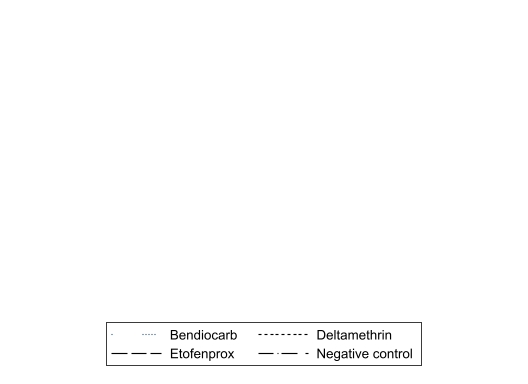


**Additional file 1: Fig. S4 Residual efficacy of three standard IRS formulations applied to four substrates, in comparison to an untreated control, measured as mortality in adult female *Anopheles gambiae* of the Kisumu strain 24 hours after exposure**

Mosquitoes were exposed to mud, cement, ceramic tile or wood treated with 0.4% bendiocarb WP, 0.025% deltamethrin WG, 0.1% etofenprox WP (manufacturers’ recommended application rates), or an untreated negative control, and mortality observed 24 hours after exposure. Values shown are average mortality in 30 mosquitoes, 10 exposed to each of three replicate tiles.
